# A Review: *Wolbachia*-Based Population Replacement for Mosquito Control Shares Common Points with Genetically Modified Control Approaches

**DOI:** 10.3390/pathogens9050404

**Published:** 2020-05-22

**Authors:** Pei-Shi Yen, Anna-Bella Failloux

**Affiliations:** Unit Arboviruses and Insect Vectors, Department of Virology, Institut Pasteur, F-75724 Paris, France

**Keywords:** mosquito control, replacement strategy, *Wolbachia*, environmental factors, arbovirus, viral adaptation

## Abstract

The growing expansion of mosquito vectors has made mosquito-borne arboviral diseases a global threat to public health, and the lack of licensed vaccines and treatments highlight the urgent need for efficient mosquito vector control. Compared to genetically modified control strategies, the intracellular bacterium *Wolbachia,* endowing a pathogen-blocking phenotype, is considered an environmentally friendly strategy to replace the target population for controlling arboviral diseases. However, the incomplete knowledge regarding the pathogen-blocking mechanism weakens the reliability of a *Wolbachia*-based population replacement strategy. *Wolbachia* infections are also vulnerable to environmental factors, temperature, and host diet, affecting their densities in mosquitoes and thus the virus-blocking phenotype. Here, we review the properties of the *Wolbachia* strategy as an approach to control mosquito populations in comparison with genetically modified control methods. Both strategies tend to limit arbovirus infections but increase the risk of selecting arbovirus escape mutants, rendering these strategies less reliable.

## 1. Half of the World’s Population Exposed to Arboviral Diseases

Mosquito-borne arboviral diseases such as chikungunya, dengue, yellow fever, and Zika have been one of the major public health issues over the last few decades, threatening more than half of the world’s population [1]. These arboviruses have dispensed with the need for amplification in wild animals to cause outbreaks in the human population. Human hosts serve simultaneously as the reservoir, amplifier, and disseminator, and the major vectors are the anthropophilic mosquitoes *Aedes aegypti* and *Aedes albopictus* [2]. Due to their complex transmission mechanisms with interactions between viruses, mosquito vectors, and vertebrate hosts, all three evolving in changing environments, the control of arboviral diseases is still extremely difficult. The frustrating lack of development of broad-spectrum vaccines against arboviruses has pointed out the importance of new alternatives for arboviral diseases control [3,4].

Chikungunya is a rapidly reemerging arboviral disease. In recent years, as the Indian Ocean lineage (IOL) evolved, this *Ae. aegypti* adapted chikungunya virus (CHIKV) has caused several outbreaks in tropical countries [5]. The newly emerged CHIKV IOL contains a mutation from Alanine to Valine at position 226 in the E1 protein, which influences the pH threshold for fusion, facilitating the virus entry [6]. Moreover, the E1-A226V mutation also increases the vector competence for *Aedes* mosquitoes, particularly for *Aedes albopictus* [7]. Therefore, the epidemic areas have extended beyond tropical regions, reaching the temperate countries in Europe where *Ae. albopictus* mosquitoes have been established since 1979 [8]; this species is now present in 20 European countries with the highest infestation levels in France [9,10] and Italy [11]. Dengue virus (DENV) alone contributes to approximately 390 million infections and tens of thousands fatal cases annually [12]. There are four DENV serotypes, namely dengue-1, -2, -3, and -4, which trigger distinct immune responses in humans [13]. Although a life-long immunity against a given DENV serotype could be acquired after a first infection, a secondary infection with another DENV serotype could facilitate dengue severe syndrome or dengue hemorrhagic fever (DHF) that increases the burden of this disease even if a secondary infection does not always necessarily lead to DHF [14,15]. Moreover, the nearly 80% of dengue asymptomatic cases enhance the risk of DENV transmission, thus complexifying the disease control. Yellow fever virus (YFV) has not yet become a global threat but heavily affects Sub-Saharan Africa and South America (mainly in Brazil), causing between 94,336 and 118,500 infection incidents annually [16]. In Africa, the urban cycle results from sporadic spillover transmission from the jungle cycle, while in America, human infections are only acquired in forests [17]. Although the YFV live-attenuated vaccine has been available since the 1930s, the global stockpile of YFV vaccines is still an issue due to the production process that requires specific pathogens-free (SPF) eggs; the limited supply of SPF eggs makes it difficult to launch an urgent vaccination campaign [18]. The recent outbreaks in Brazil and the imported cases from Angola to China have raised many concerns on the potential risk of major outbreaks, especially for the immune-naïve populations in Asia [19,20,21]. Zika virus (ZIKV) was first isolated from an infected monkey in Uganda in 1947. Starting from Yap Island in 2007 [22], a much larger outbreak in French Polynesian islands followed [23], and reached Brazil at the end of 2013 [24]. Although ZIKV infection formerly caused only mild illness and was self-limiting [22], more severe outcomes were reported, including microcephaly in newborns and the neurological affections [25,26] giving it the status of a Public Health Emergency of International Concern in 2016 by the World Health Organization (WHO). It should be noted that other emerging arboviruses have not yet caused large outbreaks; they could be the next arboviral threats owing to current global changes, including climate warming and increasing international exchanges [27]. *Aedes* mosquitoes are also transmitting Mayaro (MAYV; genus: *Alphavirus*), and Usutu (USUV; genus: *Flavivirus*) viruses that originated from South America and Sub-Saharan Africa, respectively [28,29]. Since 2000, MAYV has caused several outbreaks in South America, although the number of reported infections remains low. The spread of MAYV to non-endemic areas was reported in Europe after 2008, expanding the risk area of this disease [30]. Similar to MAYV, the USUV was first identified in 1959 [29], but the first human infection was reported at the beginning of 1981 in Africa [31]. Since then, USUV has been introduced into Europe and was repeatedly reported in mosquitoes, birds, and horses in 12 European countries [32].

## 2. Virus Overcomes Mosquito Immune Barriers to Be Transmitted by Generating Viral Quasispecies

*Aedes* mosquitoes are the major vectors in transmitting arboviruses. They have expanded their geographic distribution as a consequence of population growth, human activities, and climate change, creating conditions favorable for their proliferation and introducing the means of passive transportation [33]. Their distribution is no longer restricted to tropical regions, spreading to new geographic regions over long distances and stepping up the global impact of arboviral diseases [33].

A mosquito vector acquires an arbovirus by taking a blood meal from a viremic host, after which the virus enters into the midgut epithelial cell and replicates. After infection of the midgut, the virus needs to escape through the midgut basal lamina and disseminate into the hemocele, infecting different tissues or organs including salivary glands. Finally, in a subsequent blood meal, the virus is excreted from the salivary glands and transmitted by the mosquito bite [34].

From the midgut to the salivary glands, the virus encounters different mosquito immune responses, such as RNAi (RNA interference), the Toll, IMD (immune deficiency), and JAK-STAT (Janus kinase signal transducer and activator of transcription protein) pathways, limiting the virus infection, dissemination, and transmission [35]. Viral infections in mosquitoes result from a subtle balance between virus replication and cellular immunity, manifested by a persistent asymptomatic infection in vectors [36]. Considering the error-prone nature of viral RNA-dependent RNA polymerase, RNA viruses generate a collection of variants (namely quasispecies) during replication that facilitates viral adaptation and transmission by mosquitoes [37,38]. Thus, after passing all these filters, only a fraction of the viral population is transmitted to the vertebrate host [39]. This remains dependent on each pairing of virus-mosquito that conditions the mosquito vector competence [34].

## 3. Population Replacement Strategy to Control Mosquito-Borne Diseases

As the pivot for arboviruses transmission, the mosquito vector is considered the target for efficient arboviral diseases control. Depending on the outcomes, the mosquito population control strategies could be roughly divided into two categories, population reduction and population replacement, which by reducing the target population size or introducing an anti-pathogen phenotype into the target population, respectively, minimizes the contact between arboviruses-carrying mosquitoes and human hosts. The current strategy is to reduce the mosquito vector population with insecticides; however, in the absence of a vector surveillance program, the impact of insecticides on local biodiversity is unpredictable, in addition there is the risk in generating insecticide-resistant mosquitoes that decreases the control efficacy, calling for more species-specific alternative methods [40]. Instead of reducing the target population from the field, which might cause ecological disruption [41,42] and risk of secondary pest emergence, population replacement has been proposed as an alternative [43]. Replacing the target population with a pathogen-refractory strain could specifically reduce the pathogens’ transmission while maintaining the population in its original ecological niche, limiting the risk of secondary pest emergence [43].

A modified genetic-based population replacement approach is composed of an anti-pathogen gene and a gene-drive system, in order to suppress the pathogen replication and to spread the phenotype within the target population [44]. Different mosquito antiviral factors such as siRNA [45,46], miRNA [47,48,49], ribozymes [50,51], immune factors [52], and neutralizing antibodies [53], can act as effectors to reduce the virus infection and transmission in genetically modified mosquitoes. Combined with a proper gene-drive system, the genetically modified mosquitoes expressing a virus-refractory phenotype can replace the wild population in a few generations, that is to say in a few months for mosquitoes. Although the biosafety concerns about using genetically modified insects is still debatable [54], the highly-specific synthetic antiviral immunity used as effectors (RNAi, antiviral ribozymes, overexpressed immune genes, and neutralizing antibodies) has raised issues regarding the selection of escape virus mutants. Thus, strategies generating weaker selection in virus populations might be more sustainable [55].

## 4. *Wolbachia*-Based Mosquito Control

**Figure 1:**
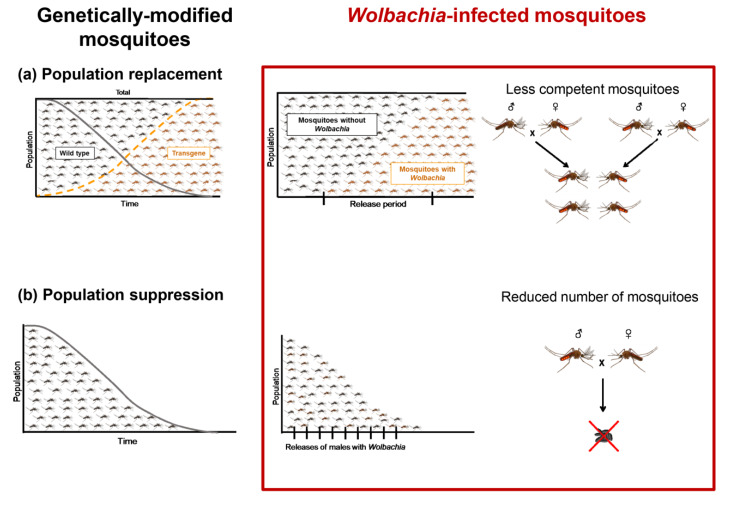
*Wolbachia*-based mosquito control strategy can be viewed as a genetically modified control approach, population replacement, or population suppression. (**a**) The population replacement strategy is to release *Wolbachia*-infected female mosquitoes that, after mating with males (*Wolbachia*-infected or not), produce viable offspring, allowing a wide spread of *Wolbachia* in the field population that harbors less competent individuals, even as the total number of mosquitoes remains unchanged. (**b**) Population suppression aims to release *Wolbachia*-infected male mosquitoes that, after mating with wild females, do not produce viable offspring, thus reducing the total number of mosquitoes.

The microbiota of mosquito vectors has a strong impact on arbovirus infections [56,57]. The endosymbiont bacteria *Wolbachia* has been in the spotlight with the discovery of its properties in suppressing the replication of vector-borne human arboviruses such as DENV, YFV, and ZIKV [58,59]. The *Wolbachia*-based insect control approach is more acceptable than the genetic modification-based approach for the public as it is a naturally existing microbe. *Wolbachia* is a Gram-negative bacterium, a member of the Alphaproteobacteria (Rickettsiales order). In arthropods, the genome of *Wolbachia* ranges from 1.2 to 1.6 Mb and contains WO prophages (named after *Wolbachia*) [60]. It was first discovered in the *Culex pipiens* mosquito in 1924 by Hertig and Wolbach [61], opening a new avenue of research owing to its high diversity and wide distribution in arthropods [62]. To date, 18 supergroups of *Wolbachia* have been identified, most of them present in arthropods [63], and more than 65% of insect species harbor *Wolbachia* [64]. Horizontal transfers of *Wolbachia* have been demonstrated between species within the same supergroup and, conversely, the same host species can host different *Wolbachia* strains. *Wolbachia* bacteria are involved in different symbiotic interactions ranging from parasitic to mutualistic [62]. Mainly transmitted vertically, it intervenes in manipulating the host reproduction in order to optimize its maternal transmission through the eggs. *Wolbachia* can induce different sex ratio distortion phenotypes in the progeny to favor females: parthenogenesis, feminization, male-killing, and cytoplasmic incompatibility (CI) [65]. In the CI phenotype, *Wolbachia*-infected females are favored over the non-infected females and males (Figure 1). The molecular mechanisms controlling the CI are today better understood: CI and its rescue are driven by toxin-antidote interactions, whose affinity between partners determines the success of the rescue [65]. For example, in *Cx. pipiens* during the first embryonic mitosis, cidA and cidB of *w*Pip are the key elements of CI traits [66], where the B factor expresses its toxic effect inducing the CI effect and the A factor acts as an antidote for the rescue. In addition to colonizing reproductive organs, *Wolbachia* bacteria are also present in somatic tissues; *Wolbachia* can be acquired from infected embryonic lineages or by passing from cell to cell [67].

## 5. *Wolbachia* in Limiting Arbovirus Transmission

*Wolbachia* can provide fitness advantages for host fertility and/or survival and can alter responses to infections to reduce arbovirus transmission. As an example, the *w*Mel *Wolbachia* strain artificially introduced in *Drosophila melanogaster* inhibited *Drosophila* C virus (DCV) infection, and *Wolbachia-*infected flies were much more resistant to DCV than the uninfected flies [68,69].

Combining their ability to invade the host population by inducing CI and to interfere negatively with the transmission of disease pathogens, *Wolbachia*-based control methods have been deployed to prevent the transmission of mosquito-borne diseases [70,71]. The bacterium *Wolbachia* has been introduced in natural populations of the mosquito *Ae. aegypti*, the urban vector of dengue, Zika, chikungunya, and yellow fever [72]. *Wolbachia* bacteria are stably maintained in field mosquito populations and attributed to the pathogen-blocking phenotype [73]. The critical question is whether this effect will persist and if adaptive changes in the mosquito vector, the bacteria, or virus may occur, hampering the success of this strategy.

*Wolbachia* can share the same niche with the virus, colonizing ovaries, gut, and salivary glands, organs that are essential for the replication and transmission of arboviruses [74]. Usually, high antiviral resistance is associated with high densities of *Wolbachia,* which could reach several hundred bacteria per cell [75] and cause a significant fitness cost (e.g., reduced fecundity, fertility, and survival) [76]. Mosquito adaptive changes may occur, leading to the evolution towards lower *Wolbachia* densities and, therefore, a reduction or loss of the antiviral phenotype. Another concern is the evolution of the virus itself; since *Wolbachia* block the replication of the virus, viral populations could be shaped to overcome such inhibition. Variants able to replicate despite the presence of *Wolbachia* could be advantaged and spread, weakening the sustainability of the *Wolbachia* control strategy. As an example, in *D. melanogaster, Wolbachia* did not present antiviral effects for the *Wolbachia*-adaptive DCV, which were genetically different from viral populations in *Wolbachia*-free controls [77]. These findings were obtained from cell cultures, which are far from replicating the conditions in a natural host, with a succession of reduction and restoration of viral diversities after crossing anatomical barriers (midgut and salivary glands) in the mosquito vector [39].

## 6. The Hypothesized Mechanisms of *Wolbachia*-Mediated Pathogen Blocking Activity

Several mechanisms have been proposed to explain the molecular basis of the pathogen-blocking phenotype: regulation of immune genes, indirect host gene regulation through other cellular machinery (RNAi, sfRNA), production of reactive oxygen species, or competition for a limited resource such as cholesterol. Growing evidence has shown that the mosquito transcriptome profiles were altered after *Wolbachia* infection [52,70,78,79]. Insect immune pathways such as Toll, IMD, and JAK/STAT pathways were activated in *Wolbachia*-infected *Ae. aegypti*, and led to an efficient reduction in replication of CHIKV, DENV, and *Plasmodium* [52,70,78,79]. In addition to the immune factors, many genes were reported to be upregulated and subsequently may be able to suppress virus replication; the genes regulating reactive oxygen species production and the upregulation of *Wolbachia*-mediated methyltransferase are also reported to suppress the replication of DENV in *Ae. aegypti* [52,80,81]. Mosquito non-coding RNA expression is also influenced upon *Wolbachia* infection, and might regulate virus replication in infected cells [82]. Although the direct interaction between virus replication and *Wolbachia*-induced miRNA is not fully understood, an extensively-expressed aae-miRNA-2940 in *Wolbachia*-infected mosquito cells was proven to upregulate methyltransferase expression and, subsequently, reduce DENV replication [81]. Moreover, this methyltransferase upregulation negatively controls the metalloprotease expression leading to a reduction in West Nile virus replication [83]. Recent studies have suggested that *Wolbachia* bacteria suppress virus replication through cellular resources allocation (e.g., intracellular space in *Wolbachia*-infected cells [70,84]), vascular trafficking, and lipid metabolism caused by *Wolbachia*-mediated cholesterol perturbation [85]. In fact, on one hand, *Wolbachia* do not have any functional lipopolysaccharide synthase and need cholesterol for membrane formation, while on the other hand, viruses rely on host cholesterol for replication. Therefore, both behave as competitors for access to cholesterol ingested by the mosquito, which is auxotrophic for this element [85]. Moreover, the limited intracellular space in *Wolbachia* infected cells is also reported to restrict DENV replication [70,84].

## 7. Viruses Evolve towards an Adaption to *Wolbachia*-Blocking Activity

**Figure 2:**
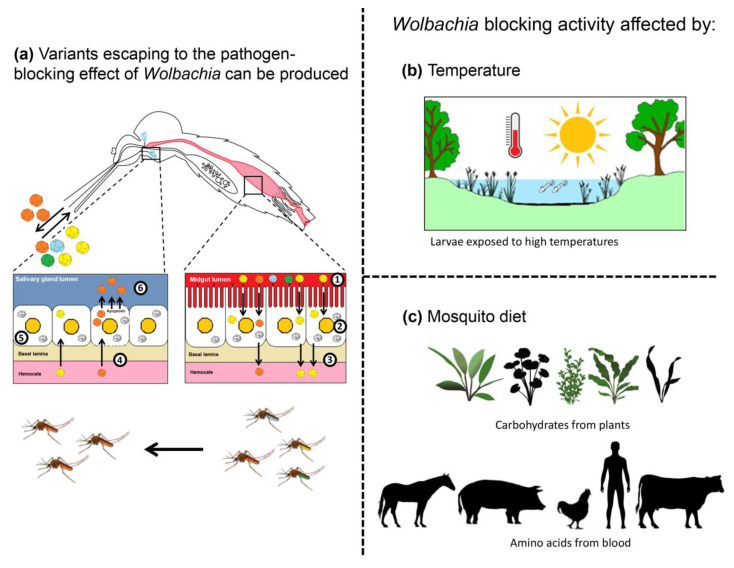
Viruses escaping from *Wolbachia* blocking activity. (**a**) After ingestion of an infectious blood meal (1), viruses enter into the midgut epithelial cells and replicate (2). Newly produced viruses are released into the hemocoel, infecting internal organs/tissues (3). After reaching the salivary glands (4), viruses replicate (5), and new viruses are excreted with saliva expectorated (6) by females when they bite. In the presence of *Wolbachia* in the midgut and salivary glands, escaping variants can be produced, leading mosquitoes to transmit viruses less sensitive to the pathogen-blocking effect. (**b**) *Wolbachia* blocking activity can be altered by environmental factors such as temperature. Mosquito larvae submitted to high temperatures produce adults with a lower density of *Wolbachia.* (**c**) Mosquito adults feeding on enriched diets (carbohydrates and amino acids) show reduced *Wolbachia* loads. Both (**b**) and (**c**) lead to a diminished blocking activity of *Wolbachia* as their bacterial densities are much lower.

Recent studies have raised concerns regarding *Wolbachia*-mediated selection pressure for arboviruses that might facilitate viral adaptive evolution and escape from pathogen-blocking effects [77]. Evidence has been brought that *Wolbachia* bacteria do not suppress the replication of viral RNA genomes in mosquito cells and, consequently, are not able to accumulate adaptive viral genomes [86,87]. The same alteration can be observed in *Wolbachia*-infected *D. melanogaster* for DCV, where *Wolbachia*-mediated selective DCV strains could be found after 10 passages in *Wolbachia*-infected flies. Despite the fact that the adapted DCV strains did not exhibit the same resistance phenotype to *Wolbachia* in newly *Wolbachia*-infected flies, evidence points to the risk of *Wolbachia*-mediated selection for virus adaptive mutations. The resulting adapted viruses might have a better chance of escaping the *Wolbachia*-mediated virus-blocking effects in drosophila [77]. Moreover, many studies have indicated the *Wolbachia* naturally infecting *Ae. albopictus*, *w*AlbA, and *w*AlbB do not show significant pathogen-blocking activity against virus infection in this mosquito [75,88,89,90,91]. Even if *w*AlbB is transinfected in other mosquito species, it cannot trigger the pathogen-blocking phenotype, as observed with other *Wolbachia* strains [92,93]. These results raise the question of the outcome of co-evolution between *Wolbachia* and viruses, highlighting the risk for viruses to escape from *Wolbachia*-mediated immunity (Figure 2a).

## 8. Environmental Factors That May Affect *Wolbachia*-Based Control Programs

### 8.1. An Abiotic Factor, the Temperature

In the field, *Wolbachia*-infected mosquitoes are exposed (naturally or not) to fluctuating and even extreme temperatures (Figure 2b). Larvae reared under cycling temperatures between 26 °C and 37 °C resulted in adults with a lower density of *Wolbachia*, an effect persisting across generations [94], and a diminished CI effect that resulted in the inability of *Wolbachia* to be transmitted vertically [95]. More interesting is the possibility that these effects could be strain-dependent. Temperature significantly affects *w*Mel and *w*MelPop-CLA in *Ae. aegypti*, whereas no effect was observed with *w*AlbB, which naturally colonizes the mosquito *Ae. albopictus*, suggesting that the *Wolbachia* adaptation might reduce the pathogen-blocking activity. Infections with *w*AlbB seem to be more applicable in a population reduction strategy than pathogen suppression in field conditions. The duration of heat stress also alters *Wolbachia* density [96]. A long duration of heat stress of *Ae. aegypti* immature stages leads to lower *w*Mel densities in adults. Complementary studies using local mosquitoes and field temperatures are required in *Wolbachia*-released sites [97].

Temperature might influence *Wolbachia* density in *Ae. aegypti* through bacteriophage WO infection [98]. High temperatures may reduce *Wolbachia* densities in *Ae. aegypti* through interactions with WO, which infects *w*Mel [99] and *w*AlbB [100]. This phage undergoes cycles of lysogenic and lytic phases; heat shock triggers the lytic phase during which the phage replicates and causes *Wolbachia* lysis, reducing its densities [101]. However, the temperature has a positive effect on virus replication with significantly higher titers of DENV at temperature > 28 °C in *Ae. aegypti* mosquitoes at day 10 post-infection in salivary glands [102]. More globally, high temperature is likely to weaken the *Wolbachia*-mediated pathogen-blocking activity by shortening the extrinsic incubation period (the time necessary for mosquitoes to become infectious after an infectious blood meal) [103,104,105], and reducing *Wolbachia* density through bacteriophage WO.

### 8.2. A Biotic Factor, the Host Diet for Mosquito Vectors

Host diet notably influences *Wolbachia* load (Figure 2c) [106]. Flies reared with yeast-enriched diets show reduced *Wolbachia* loads in the host female germline, and flies with a high sucrose diet show an increase of *Wolbachia* titer in oocytes. Thus, *Wolbachia* bacteria rely upon host uptake of amino acids and carbohydrates. Exposure to yeast-enriched food alters *Wolbachia* nucleoid morphology in oogenesis [106]. The yeast-induced *Wolbachia* depletion is mediated by the somatic target of rapamycin (TOR) and insulin signaling pathways. These findings are critical in programs combating arboviral diseases by releasing *Wolbachia*-infected vectors [73,107]. Thus, the natural environment including host diet (i.e., nature of blood and sugar absorbed by released mosquitoes) should be considered when evaluating the efficiency of *Wolbachia* strategies in the field.

## 9. Obstacles for Population Replacement Program

### 9.1. Unclear Wolbachia Distribution in Wild Mosquito Populations

*Ae. aegypti* populations were thought to be free of naturally-harboring *Wolbachia*; as a result, many *Wolbachia*-based mosquito control strategies were deployed worldwide to reduce or replace the target mosquito populations via the CI nature of *Wolbachia*. However, accumulating evidence shows the existence of naturally-occurring *Wolbachia* in wild *Ae. aegypti* populations in India [108], Malaysia [109], Panama [110], Thailand [111], Philippines [112], and the United States [113,114,115]. Even though only small proportions of *Ae. aegypti* were reported in naturally-harboring *Wolbachia* in field-collected populations, the presence of naturally-infecting *Wolbachia* tends to increase the instability for replacing the target populations, thus weakening the efficacy of mosquito control programs.

### 9.2. The Robust CI Effect Limits the Solutions for Wolbachia Re-Replacement

On the one hand, the naturally existing *Wolbachia* in the target population might be an obstacle for the mosquito population replacement program. On the other hand, the robust CI effect also increases the difficulty to re-replace the target population that has been already replaced by *Wolbachia*-harboring mosquitoes. Because of this, the *Wolbachia*-mediated pathogen-blocking strategy implemented can deviate from the objectives initially planned. Thus, a more flexible replacement program is required to adapt to this scenario. The results of computational modeling have suggested that unless a correct *Wolbachia* strain is used, the selection could favor the strain with a lower fitness cost (commensal infection), thus replacing the existing *Wolbachia*-harboring population. Furthermore, the second replacement can be less efficient than the first one [116].

## 10. Conclusions

Mosquito control, as an essential step for mosquito-borne diseases transmission management, either by reducing the target population size or replacing the target population with a pathogen-refractory strain, could efficiently reduce the contact between mosquitoes and hosts, thereby interrupting the disease transmission. Compared to a genetically modified control strategy, a *Wolbachia*-based control strategy is considered a promising alternative to control mosquito populations due to its natural and environmentally friendly features: It can carry a broad pathogen-blocking activity and robust CI effect, which together ensure the efficacy in reducing mosquito-borne diseases transmission. However, complex interactions and resulting co-evolution processes among mosquito, virus, host, and the environment, make difficult any mosquito-borne disease control strategies. Thus, monitoring and prevention programs to avoid escape mutants in viral populations must be attentively planned if the targeted objective is to reach a sustainable control strategy.
